# Association between night shift work and NAFLD: a prospective analysis of 281,280 UK Biobank participants

**DOI:** 10.1186/s12889-023-16204-7

**Published:** 2023-07-03

**Authors:** Hangkai Huang, Zhening Liu, Jiarong Xie, Chengfu Xu

**Affiliations:** 1 Department of Gastroenterology, Zhejiang Provincial Clinical Research Center for Digestive Diseases, The First Affiliated Hospital, Zhejiang University School of Medicine. No, 79 Qingchun Road, Hangzhou, 310003 China; 2 Department of Gastroenterology, Ningbo First Hospital, Ningbo, China

**Keywords:** Night shift, Nonalcoholic fatty liver disease, Genetic risk

## Abstract

**Context:**

This study aimed to investigate the association between night shift work and the risk of nonalcoholic fatty liver disease (NAFLD).

**Methods:**

We conducted a prospective analysis of 281,280 UK Biobank participants. Cox proportional hazards models were used to estimate the association of night shift work with incident NAFLD. Polygenic risk score analyses were performed to assess whether a genetic predisposition to NAFLD modified the association.

**Results:**

During a median follow-up of 12.1 years (3,373,964 person-years), 2,555 incident NAFLD cases were identified. Compared with workers who never/rarely worked night shifts, those who worked some night shifts or usual/permanent night shifts were 1.12 (95% CI: 0.96–1.31) and 1.27 (95% CI: 1.08–1.48) times more likely to develop NAFLD, respectively. Among the 75,059 participants who had reports on lifetime experience of night shift work, those with a longer duration, a higher frequency, more consecutive night shifts and a longer length per shift all showed higher risks of incident NAFLD. Further analyses showed that the association between night shift work and incident NAFLD was not modified by a genetic predisposition to NAFLD.

**Conclusions:**

Night shift work was associated with increased risks of incident NAFLD.

**Supplementary Information:**

The online version contains supplementary material available at 10.1186/s12889-023-16204-7.

## Introduction

Nonalcoholic fatty liver disease (NAFLD) is one of the most common chronic liver diseases and poses a serious challenge to global public health [1]. The global prevalence of NAFLD is increasing rapidly and reached 29.8% in 2019 [2]. The economic burden of NAFLD is projected to be substantial, with an estimated annual direct cost of 103 billion dollars in the United States [3]. NAFLD is associated with a higher risk of liver-related complications, extrahepatic cancers, cardiovascular disease and all-cause mortality [4, 5]. NAFLD was associated with a 1.93-fold higher risk of overall mortality, and the risk increased gradually with the histological progression of NAFLD [6]. The early identification and modification of risk factors may be critical in the prevention of NAFLD, which ultimately can ease the disease-related health resource burden.

With industrialization and economic growth, shift work has become increasingly common [7]. Shift work refers to labor conducted outside conventional daytime hours, including daily, nightly, rotating or irregular shift work. Nineteen percent of employees participated in night shift work at least once per month [8]. Individuals who engage in shift work, particularly night shift work, suffer from altered sleep timing and shifted mealtimes and are exposed to light at night [9], resulting in a disrupted circadian rhythm. Accumulating evidence suggests that night shift work is associated with a series of cardiometabolic diseases. Night shift workers were reported to be at higher risks of coronary heart disease [10], atrial fibrillation [11] and type 2 diabetes [12]. However, whether night shift work is associated with NAFLD needs to be explored in well-designed and large-scale longitudinal studies.

In addition to environmental and lifestyle factors, genetics also play a role in the risk of NAFLD. A genome-wide association study (GWAS) identified 12 loci associated with NAFLD in 2020 [13]. Since genetic susceptibility may modify the relationship between environmental and lifestyle factors and metabolic disease, it is important to explore the interactive effect between genetics and night shift work on NAFLD.

In this study, we analyzed whether night shift work was associated with NAFLD risks. Furthermore, we explored whether a longer duration and higher frequency of night shift work and a longer length of each night shift were associated with higher risks of incident NAFLD and whether this association was modified by genetic predisposition.

## Materials and methods

### Study population

The UK Biobank recruited over 500,000 participants aged 37 to 73 years from 22 assessment research centers across England, Scotland, and Wales between April 2007 and July 2010. All participants completed a touch screen questionnaire, underwent a range of physical measures and provided biological samples at baseline. Details of the study design and data processing have been described elsewhere [12].

**Fig. 1:**
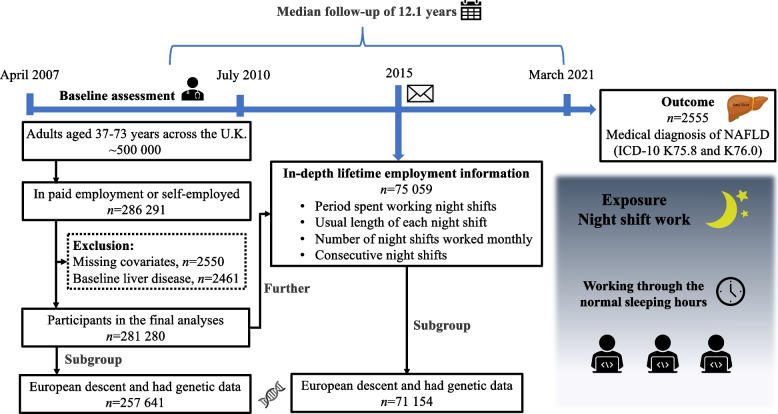
Summary of study design and analytical strategy

We restricted our analyses to participants with paid employment or who reported self-employment at baseline (*n* = 286,291). Among those individuals, we excluded those with missing data for covariates or who had liver disease at baseline (Supplementary Table S1). Overall, 281,280 participants were included. Furthermore, 75,059 participants provided in-depth lifetime employment information by completing an online follow-up questionnaire in 2015. To further explore whether the association between night shift and NAFLD was modified by genetic predisposition, we extracted participants with available genetic data from the included participants (Fig. 1). The UK Biobank study was approved by the National Health Service National Research Ethics Service (ref. 11/NW/0382), and all participants provided written informed consent.

### Exposure

Shift work is a work arrangement that is not performed during normal daytime hours (9 a.m. to 5 p.m.), which means working afternoons, evenings or nights or rotating through these kinds of shifts. If participants indicated they had paid employment or were self-employed, they were then asked "*Does your work involve shift work*?" (Data-Field 826). Those who answered ‘*sometimes, usually, and always*’ were further asked whether their work involved night shift work that ‘*is a work schedule involving working through the normal sleeping hours, for instance working through the hours from 12 a.m. to 6 a.m.*’ (Data-Field 3426). Based on those two questions, we categorized participants’ current night shift work status as “never or rarely night shifts,” “some night shifts” and “usual or permanent night shifts”.

In the lifetime employment assessment, participants reported information about the number of years in each job and the details of night shifts that each job entailed. The lifetime employment information before baseline was used for each participant. Combining those fields, we calculated the (i) duration (i.e., number of years with night shifts), (ii) frequency (i.e., the average number of night shifts per month), (iii) length (i.e., the usual length (hours) of each night shift) of night shift work, and (iv) consecutive night shifts during night shift periods.

### Outcomes

Data on the date and cause of hospital admissions were identified through linked hospital admissions data, including Hospital Episode Statistics–Admitted Patient Care (England), Scottish Morbidity Records–General/Acute Inpatient and Day Case Admissions (Scotland) or Patient Episode Database for Wales. The date and cause of death were obtained through death certificates held by the NHS Information Centre (England and Wales) and the NHS Central Register (Scotland).

Petermann-Rocha et al*.* published a research article on the topic of grip strength and NAFLD using data from the UK Biobank earlier [14]. The definition of NAFLD in this analysis was learned from their study. We diagnosed NAFLD as a hospital admission or death with ICD-10 (international classification of diseases, 10^th^ revision) codes K76.0 (fatty [change of] liver, not elsewhere classified) and K75.8 (other specified inflammatory liver diseases). Follow-up time was calculated from the date of attendance to the time of NAFLD diagnosis, death, or the end of follow-up (March 2021), whichever occurred first.

### Genotyping and SNP selection

Participants in the UK Biobank were genotyped using the custom UK BiLEVE array or the UK Biobank Axiom array. Twelve single-nucleotide polymorphisms (SNPs) representative of loci were associated with NAFLD based on a GWAS of Europeans, of which six SNPs were excluded based on a linkage disequilibrium level of *r*^*2*^ < 0.001 and distance window of 10,000 kb (Supplementary Table S2). The polygenic risk score (PRS) is an estimate of an individual’s genetic susceptibility to a disease [15]. We then created a PRS by computing the sum of risk alleles that an individual has, weighted by the risk allele effect sizes as estimated by the GWAS mentioned above, according to the guide provided by Collister et al*.* [16]. We then determined whether participants of European descent were at high (highest quartile), intermediate (mid-two quartiles), or low (lowest quartile) genetic risk for NAFLD.

### Covariates

Information on potential confounders, including age (continuous), sex, ethnicity (white or others), Townsend deprivation index (continuous, reflecting socioeconomic status), education level (university/college degree or others), household income (less than £18,000, £18,000 to £30,999, £31,000 to £51,999, £52,000 to £100,000, greater than £100,000, or do not know/prefer not to answer), self-reported smoking status (never, former or current smoker), self-reported frequency of alcohol intake (daily/almost daily, 1–4 times a week, 1–3 times a month, or special occasions only/never), and physical activity level [< 600 (inadequate), 600–3000 (moderate), > 3000 (vigorous) metabolic equivalent of energy (MET) minutes per week, or missing], was also assessed. Hypertension was defined as systolic blood pressure level of 140 mmHg or more and/or diastolic blood pressure level of 90 mmHg or more, use of medications for blood pressure or self-reported or diagnosed by a doctor [17]. Diabetes was defined as blood glucose ≥ 11.1 mmol/L, glycated hemoglobin (HbA_1_c) ≥ 48 mmol/mol, under insulin treatment or self-reported or diagnosed by a doctor [18].

### Statistical analyses

Cox proportional hazards models were used to estimate the hazard ratios (HRs) and 95% confidence intervals (CIs) for the associations between current and lifetime night shift variables and incident NAFLD. We checked the proportional hazards assumption by plotting the log–log survival curves. Missing data were treated as an individual category for variables with a missing rate of > 5% (i.e., physical activity). Current shift work status was categorized as never or rarely night shifts, some night shifts, or usual/permanent night shifts. For participants who reported lifetime employment, we assessed associations of cumulative night shift work duration (none, < 10 years, or ≥ 10 years), average monthly frequency of night shifts (none, < 8 night shifts/month, or ≥ 8 night shifts/month), average length of each night shift (none, < 8 h, 8–12 h, or > 12 h), and consecutive night shifts during night shift periods (none, 1 shift, 2–5 shifts, or > 5 shifts) with NAFLD odds.

Three models with increasing adjustment were utilized. Model 1 was adjusted for age, sex, and ethnicity. Model 2 was adjusted for model 1 plus socioeconomic factors (Townsend deprivation index, education level, and household income) and lifestyle factors (self-reported smoking status, self-reported frequency of alcohol intake, and physical activity level). Model 3 was further adjusted for baseline hypertension, diabetes and PRS for NAFLD. In addition, *P* values for trend were calculated when the variables related to night shift work described above were treated as continuous values and tested using the Wald test.

As body mass index (BMI) may be a potential mediator of the association between night shift work and NAFLD, we also conducted a mediation model adjusting for BMI separately in addition to model 3 and calculated the mediation effect using the SAS macro *%mediate*.

We then examined whether the association of night shift work with NAFLD could be modified by genetic risk. Hence, we included an interaction term in the regression model. The hazard ratio of the product term was the measure of interaction on the multiplicative scale, and the Wald test was used to evaluate whether this term was statistically significant. In addition, we estimated the relative excess risk due to interaction (RERI) and its corresponding 95% confidence intervals to test the statistical significance of the additive interactions [19].

We further verified the robustness of the results by excluding the first 2 years of follow-up (to avoid reverse causality) and participants with poor sleep patterns (score 0 or 1) [20] or excessive alcohol intake (> 30 g/d for men or > 20 g/d for women) and widening the definition of endpoints, including liver fibrosis, cirrhosis and its complications (K74.0, K74.1, K74.2, K74.6, K76.6, K76.7, I85.0, I85.9, I86.4, I98.2, I98.3, R18, Z94.4), to see whether the associations were fundamentally changed. We also conducted subgroup analyses stratified by age, sex, household income, Townsend deprivation index, education level, smoking status, physical activity, hypertension, and diabetes.

All analyses were performed using SAS version 9.4 (SAS Institute, Cary, NC). We considered two-sided *P* values < 0.05 to be significant.

## Results

### Population characteristics

The baseline characteristics of the study population by current shift work schedule are shown in Table 1. In general, among 281,280 participants, night shift workers were more likely to be younger, male, nonwhite, less educated, and more physically active and to have lower socioeconomic status and income. In addition, they tended to be current smokers and had a higher BMI but consumed less alcohol daily. Hypertension and diabetes were more prevalent among night shift workers. Characteristics by the duration of lifetime night shift work are presented in Supplementary Table S3. Similar patterns were discovered in the subsamples categorized according to lifetime employment history.

**Table 1:** UK Biobank participants’ characteristics by current night shift work exposure

Variables	Current work schedule			*P* value
	Never/rarely night shifts (*n* = 256,605)	Some night shifts (*n* = 13,857)	Usual/permanent night shifts (*n* = 10,818)	
Male (%)	46.63	62.08	62.22	< 0.001
Age (years)	52.9 ± 7.09	51.19 ± 6.86	51.31 ± 6.81	< 0.001
White (%)	94.56	87.58	86.65	< 0.001
Townsend deprivation index	-1.43 ± 2.96	-0.55 ± 3.31	-0.42 ± 3.31	< 0.001
College or university degree (%)	39.16	23.68	15.31	< 0.001
Household income (£)				< 0.001
< 18,000	9.13	11.64	12.99	
18,000 to 30,999	19.3	23.8	27.54	
31,000 to 51,999	28.06	30.4	31.87	
52,000 to 100,000	26.34	19.56	15.46	
> 100,000	7.04	3.95	1.58	
Physical activity (%)				< 0.001
Inadequate	16.82	10.7	9.61	
Moderate	42.81	32.59	29.07	
Vigorous	23.24	35.18	36.67	
Smoking status (%)				< 0.001
Never	57.91	52.93	52.81	
Previous	31.98	30.78	30.14	
Current	10.11	16.29	17.05	
Alcohol consumption (%)				< 0.001
Never or special occasions only	15.57	20.36	24.17	
1 to 3 times/month	11.68	13.16	14.27	
1 to 4 times/week	52.58	50.05	49.75	
Daily or almost daily	20.17	16.42	11.81	
Body mass index (kg/m^2^)	27.15 ± 4.67	28.19 ± 4.89	28.41 ± 4.9	< 0.001
Hypertension (%)	46.95	49.83	51.12	< 0.001
Diabetes (%)	4.04	5.50	6.25	< 0.001

Values are the mean (± standard deviation, SD) or percentage (%) and were examined by one-way ANOVA or chi-square test

### Associations of current night shift work with incident NAFLD

During a median follow-up of 12.1 years (3,373,964 person-years), 2,555 incident NAFLD cases were recorded, and the incidence rate increased across the night shift work schedule categories (72.85/100,000 person-years for never/rarely night shifts, 97.31/100,000 person-years for some night shifts, and 116.61/100,000 person-years for usual/permanent night shifts). We next constructed several regression models to examine the association of night shift work with the risk of NAFLD (Table 2). In age-, sex-, and ethnicity-adjusted model 1, compared with individuals who never/rarely worked the night shift, those with some night shifts or usual/permanent night shifts had 1.35 (1.15–1.58) and 1.61 (1.36–1.90) higher risks of NAFLD, respectively. This association remained significant in usual/permanent night shift workers after adjusting for more socioeconomic and lifestyle factors in model 2 (HR: 1.32, 95% CI: 1.12–1.56) and model 3 (HR: 1.27, 95% CI: 1.08–1.48). We then conducted a mediation analysis and discovered that BMI mediated 31.7% (95% CI: 14.3–56.4, *P* < 0.001) of this association. These results indicate that current night shift work is a risk factor for the development of NAFLD.

**Table 2:** Current night shift work and NAFLD odds in the UK Biobank

Variables	Never/rarely night shifts	Some night shifts	Usual/permanent night shifts	*P*_trend_	Mediation effect (%) (95% CI)
Case/Sample	2243/256605	161/13857	151/10818		
Incidence rate (/100,000 person years)	72.85	97.31	116.61		
Model 1	1.00 (ref)	1.35 (1.15–1.58)	1.61 (1.36–1.90)	< 0.001	
Model 2	1.00 (ref)	1.17 (1.00–1.38)	1.32 (1.12–1.56)	< 0.001	
Model 3	1.00 (ref)	1.12 (0.96–1.31)	1.27 (1.08–1.48)	0.003	
Mediation model	1.00 (ref)	1.04 (0.89–1.22)	1.19 (1.01–1.39)	0.050	31.7 (14.3–56.4) *P* < 0.001

Cox proportional hazards regression models for NAFLD were performed

Model 1 was adjusted for age, sex, and ethnicity (white or others)

Model 2 was model 1 plus further adjustment for Townsend deprivation index, education level (university/college degree or others), household income (less than £18,000, £18,000 to £30,999, £31,000 to £51,999, £52,000 to £100,000, greater than £100,000, or do not know/prefer not to answer), self-reported smoking status (never, former or current smoker), self-reported frequency of alcohol intake (daily/almost daily, 1–4 times a week, 1–3 times a month, or special occasions only/never), and physical activity level (< 600, 600–3000, > 3000 MET minutes per week, or missing)

Model 3 was model 2 plus further adjustment for diabetes, hypertension, and PRS for NAFLD

The mediation model was adjusted for BMI separately in addition to model 3, and the mediation effect was calculated

### Associations of lifetime night shift work with incident NAFLD

We then examined associations between lifetime night shift work (duration, frequency, length, and consecutiveness) and NAFLD risk (*n* = 75,059; 502 incident cases). Overall, compared with individuals who never worked night shifts, those who had longer night shift durations, more frequent night shifts, longer lengths per shift, and more consecutive night shifts were more likely to develop NAFLD [incidence rate (/100,000 person years): 48.29 in control, 87.86 in ≥ 10 years, 87.94 in ≥ 8 night shifts/month, 85.02 in > 12 h/shift, and 83.74 in > 5 consecutive night shifts]. Furthermore, even after multivariable adjustment, these parameters were significantly associated with a higher risk of NAFLD [HR (95% CI): 1.51 (1.20–1.91) in ≥ 10 years, 1.45 (1.15–1.83) in ≥ 8 night shifts/month, 1.53 (1.17–2.00) in > 12 h/shift, and 1.50 (1.12–2.01) in > 5 consecutive night shifts, all *P*_*trend*_ < 0.01]. Detailed figures are displayed in Tables 3, 4, 5. In addition, BMI was discovered to be a mediator in the association of these parameters with NAFLD. These findings strengthen the links between night shift work and NAFLD.

**Table 3:** Lifetime duration of night shift work and risk of NAFLD

Variables	Lifetime duration of night shift work			*P*_trend_	Mediation effect (%) (95% CI)
	None	< 10 years	≥ 10 years		
Case/Sample	333/57003	82/9843	87/8213		
Incidence rate (/100,000 person years)	48.29	68.95	87.86		
Model 1	1.00	1.40 (1.10–1.79)	1.77 (1.40–2.25)	< 0.001	
Model 2	1.00	1.30 (1.02–1.66)	1.55 (1.22–1.98)	< 0.001	
Model 3	1.00	1.23 (0.97–1.55)	1.51 (1.2–1.91)	< 0.001	
Mediation model	1.00	1.15 (0.91–1.45)	1.34 (1.06–1.68)	0.019	28.9 (14.8–48.8) *P* < 0.001

Model 1 was adjusted for age, sex, and ethnicity (white or others)

Model 2 was model 1 plus further adjustment for Townsend deprivation index, education level (university/college degree or others), household income (less than £18,000, £18,000 to £30,999, £31,000 to £51,999, £52,000 to £100,000, greater than £100,000, or do not know/prefer not to answer), self-reported smoking status (never, former or current smoker), self-reported frequency of alcohol intake (daily/almost daily, 1–4 times a week, 1–3 times a month, or special occasions only/never), and physical activity level (< 600, 600–3000, > 3000 MET minutes per week, or missing)

Model 3 was model 2 plus further adjustment for diabetes, hypertension, and PRS for NAFLD

The mediation model was adjusted for BMI separately in addition to model 3, and the mediation effect was calculated

**Table 4:** Average lifetime night shift frequency and risk of NAFLD

Variables	Average lifetime night shift frequency			*P*_trend_	Mediation effect (%) (95% CI)
	None	< 8/month	≥ 8/month		
Case/Sample	333/57003	81/9778	88/8278		
Incidence rate (/100,000 person years)	48.29	68.72	87.94		
Model 1	1.00	1.41 (1.10–1.79)	1.77 (1.39–2.24)	< 0.001	
Model 2	1.00	1.32 (1.03–1.68)	1.53 (1.20–1.95)	< 0.001	
Model 3	1.00	1.27 (1.01–1.61)	1.45 (1.15–1.83)	< 0.001	
Mediation model	1.00	1.18 (0.94–1.5)	1.29 (1.02–1.63)	0.018	29.6 (14.0–52.1) *P* < 0.001

Model 1 was adjusted for age, sex, and ethnicity (white or others)

Model 2 was model 1 plus further adjustment for Townsend deprivation index, education level (university/college degree or others), household income (less than £18,000, £18,000 to £30,999, £31,000 to £51,999, £52,000 to £100,000, greater than £100,000, or do not know/prefer not to answer), self-reported smoking status (never, former or current smoker), self-reported frequency of alcohol intake (daily/almost daily, 1–4 times a week, 1–3 times a month, or special occasions only/never), and physical activity level (< 600, 600–3000, > 3000 MET minutes per week, or missing)

Model 3 was model 2 plus further adjustment for diabetes, hypertension, and PRS for NAFLD

The mediation model was adjusted for BMI separately in addition to model 3, and the mediation effect was calculated

**Table 5:** Average length of each night shift and risk of NAFLD

Variables	Average length of each night shift during night shift periods				*P*_trend_	Mediation effect (%) (95% CI)
	None	< 8 h	8–12 h	> 12 h		
Case/Sample	333/57003	48/5982	62/6337	59/5737		
Incidence rate (/100,000 person years)	48.29	66.63	81.03	85.02		
Model 1	1.00	1.31 (0.97–1.78)	1.67 (1.27–2.18)	1.74 (1.32–2.3)	< 0.001	
Model 2	1.00	1.18 (0.87–1.60)	1.47 (1.11–1.93)	1.61 (1.22–2.13)	< 0.001	
Model 3	1.00	1.17 (0.87–1.57)	1.38 (1.06–1.80)	1.53 (1.17–2.00)	< 0.001	
Mediation model	1.00	1.06 (0.79–1.42)	1.27 (0.97–1.66)	1.38 (1.06–1.81)	0.004	24.2 (12.0–42.7) *P* < 0.001

Model 1 was adjusted for age, sex, and ethnicity (white or others)

Model 2 was model 1 plus further adjustment for Townsend deprivation index, education level (university/college degree or others), household income (less than £18,000, £18,000 to £30,999, £31,000 to £51,999, £52,000 to £100,000, greater than £100,000, or do not know/prefer not to answer), self-reported smoking status (never, former or current smoker), self-reported frequency of alcohol intake (daily/almost daily, 1–4 times a week, 1–3 times a month, or special occasions only/never), and physical activity level (< 600, 600–3000, > 3000 MET minutes per week, or missing)

Model 3 was model 2 plus further adjustment for diabetes, hypertension, and PRS for NAFLD

The mediation model was adjusted for BMI separately in addition to model 3, and the mediation effect was calculated

### Interactions between night shift work and polygenic risk score on NAFLD risk

Gene–environment interactions play an important role in the progression of diseases. Here, we also analyzed whether this interaction exists in the association of night shift work with NAFLD. First, we calculated a weighted PRS individually among participants (*n* = 257,641) of European descent according to a genome-wide association study (GWAS) of Europeans. As expected, a higher genetic risk for NAFLD was associated with a higher likelihood of NAFLD (Supplementary Fig. 1a, *P*_*trend*_ < 0.001). However, a multiplicative interaction was not observed between night shift work and the PRS (*P*_*interaction*_ = 0.206 for current night shift status, *P*_*interaction*_ = 0.385 for lifetime duration, *P*_*interaction*_ = 0.151 for frequency of night shift work, *P*_*interaction*_ = 0.184 for average length of each night shift, *P*_*interaction*_ = 0.386 for consecutive night shifts during night shift periods). Similarly, additive interactions (reflected by the RERI) were also not found (Supplementary Fig. 1b–f).

### Subgroup analyses and sensitivity analyses

Supplementary Tables S5, S6, S7, S8, S9 show the results stratified by age, sex, income, Townsend deprivation index, education level, smoking, physical activity, hypertension, and diabetes. The interaction effect between sex and average length of each night shift on the risk of NAFLD was significant (_HR>12 h *vs* none_ (95% CI): 2.20 (1.57–3.07) for males and 0.88 (0.54–1.42) for females, *P*_*interaction*_ = 0.012, Supplementary Table S8).

In sensitivity analyses (Supplementary Table S10), after excluding people with incident NAFLD for < 2 years, excessive alcohol intake, and poor sleep pattern or widening the definition of endpoint, the results did not substantially differ from those observed in our aforementioned analyses.

## Discussion

In this large-scale cohort study, we found that night shift work was significantly associated with an increased risk of incident NAFLD. First, individuals who worked night shifts had a significantly higher risk of incident NAFLD than those who never/rarely worked night shifts. Second, a longer duration, a higher frequency, more consecutive night shift work and longer hours per night shift were associated with higher risks of NAFLD, and the risk gradually increased with the progression of these traits. Third, the association between night shift work and incident NAFLD remained significant irrespective of genetic predisposition to NAFLD.

Compelling evidence suggests that night shift work is associated with cardiometabolic disease [21–29]. A prospective cohort study showed that working at least three night shifts per month was associated with higher risks of coronary heart disease [10]. Another cohort involving 0.28 million participants reported that usual or permanent night shifts were associated with a 1.22-fold risk of incident coronary heart disease over 10 years of follow-up [11]. An association between night shift work and type 2 diabetes was also found in a cohort study of 0.14 million nurses [30]. Notably, these studies all focused on the duration and frequency of night shift work and demonstrated that long durations and higher frequencies had greater adverse effects on cardiovascular health. In 2020, a cross-sectional study of 6,881 steel production workers from China revealed that current night shift work was associated with a 1.23-fold risk of ultrasonography-diagnosed NAFLD [31]. In line with the findings of that cross-sectional study, our long-term prospective analysis using UK Biobank data found that night shift work was positively associated with the risk of incident NAFLD. Moreover, we found that lifetime duration, frequency, consecutive times of night shifts and average length of each shift were all linearly associated with the risks of incident NAFLD.

The strengths distinguishing our study from the previous one are as follows. First, our study was a cohort study with long-term follow-up that identified incident NAFLD cases rather than a cross-sectional study. Second, our study is a large-scale cohort study involving 0.28 million workers with current shift work reports and 0.07 million workers with lifetime shift work reports. Third, we assessed for the first time whether genetic predisposition modified the association between night shift work and NAFLD. We also analyzed the interaction between NAFLD and age, sex, smoking, physical activity, hypertension and diabetes on NAFLD and found that there was no evidence of multiplicative or additive interactions between genetic predisposition for NAFLD and night shift work on the risks of NAFLD.

The potential mechanism linking night shift work and NAFLD remains unclear, but there are several possible explanations. Night shift work is characterized by altered sleep-activity patterns, shifted eating habits and exposure to light at night, causing circadian misalignment [8]. Animal models have imitated these conditions to mimic night shift work in humans. Mice with chronic lighting-induced clock disruption showed glucose intolerance and insulin resistance [32]. The insulin-induced phosphorylation of Akt in white adipose tissue was abolished, and the diurnal regulation of insulin sensitivity was disrupted [33]. In addition, the diurnal expression of genes involved in the inflammatory response was disrupted, and an increased number of macrophages infiltrated white adipose tissue. The serum levels of proinflammatory indexes, including tumor necrosis factor-α, C-reactive protein and interleukin-6, were also elevated [34]. In addition, oxidative stress damage was more severe in the shift group [35].

This study has public health implications for the prevention of NAFLD. Since compelling evidence suggests that unhealthy lifestyles and metabolic conditions such as smoking, lack of physical activity, obesity, diabetes and dyslipidemia are risk factors for NAFLD, management of these factors is regarded as a critical strategy for NAFLD prevention [36]. Our findings indicated that reducing the duration and frequency of night shift work may be valuable in preventing the development of NAFLD. Given the increasingly common phenomenon of night shift work, interventions on work schedules may be beneficial, especially for those who are already at high risk of NAFLD.

In this study, BMI was identified as a mediator in the positive association between night shift work and incident NAFLD. Previous studies reported that a longer duration and higher frequency of night shift work were associated with increased risks of overweight and obesity [37–40]. A close relationship of BMI with the development of NAFLD was also revealed [41]. The increased risk of NAFLD among individuals with night shift work was partly attributable to an increase in BMI. We speculated that cases of NAFLD may be prevented by weight control, especially in night shift workers. In addition, there was a significant interactive effect between sex and the length of night shifts on the risk of NAFLD. Compared with female workers, male workers with a longer length of each shift showed higher odds of developing NAFLD. This was consistent with a previous cross-sectional study that investigated this association [31]. More attention should be given to screening for NAFLD among male night-shift workers considering that they are at higher risk of NAFLD.

This study has several limitations. First, this observational study analyzed the prospective association of night shift work with NAFLD risks. However, whether night shift work directly causes NAFLD remains to be determined. Second, although known confounding variables have been adjusted, unmeasured potential confounding factors may still exist. Third, we identified NAFLD from hospital admission records and death records. The incidence rate of NAFLD in this study was 75.73/100,000 person-years, a considerably smaller rate than that reported in the general population [42]. When analyzing data retrieved from the UK Biobank, we were unable to identify the severity of NAFLD due to insufficient data for calculating the model for end-stage liver disease score. Fourth, The exposure was extracted from questionnaires that included whether participants were involved in shift work and the duration, frequency and length of shift work. We were unable to measure the exposures as accurately as laboratory parameters. Fifith, the participants recruited by the UK Biobank are not representative of the UK general population in terms of socioeconomic characteristics and lifestyles. Risk estimates can be generalized, summary estimates such as prevalence and incidence should not be.

In conclusion, night shift work, particularly night shift work with long duration and high frequency, was associated with an increased risk of NAFLD. There should be a more comprehensive assessment of the health impact of night shift work.

## Supplementary Information

**Additional file 1: Table S1.** Criteriafor the liver disease at baseline. **Table S2. **Characteristics of 6 NAFLD-associated SNPs in the UK Biobank. **Table S3. **UK Biobank participants characteristics by lifetime cumulativenight shift work duration. **Table S4.** Average consecutive night shifts and risk of NAFLD. **Table S5. **Subgroup analyses in current night shift work and NAFLD. **Table S6. **Subgroup analyses in lifetime duration of night shift work and NAFLD. **Table S7. **Subgroup analyses in average lifetime night shift frequency and NAFLD. **Table S8. **Subgroup analyses in average length of each night shift and NAFLD. **Table S9. **Subgroup analyses in average consecutive night shifts and NAFLD.** Table S10.** Sensitivity analysis. **Figure S1.** Associations of night shift work with incident NAFLD by genetic risk.

## Data Availability

The datasets analyzed during the current study are available in the UK Biobank repository, http://ukbiobank.ac.uk/register-apply/.

## Abbreviations

BMI: Body mass index
CIs: Confidence intervals
GWAS: Genome-wide association study
HRs: Hazard ratios
ICD-10: International classification of diseases
NAFLD: Nonalcoholic fatty liver disease
PRS: Polygenic risk score
RERI: Relative excess risk due to interaction
SNPs: Single-nucleotide polymorphisms
