# Supplementary material for: Association between night shift work and NAFLD: a prospective analysis of 281,280 UK Biobank participants
Source: BMC Public Health. 2023 Jul 3;23:1282. doi: 10.1186/s12889-023-16204-7 (PMC10318710; doi:10.1186/s12889-023-16204-7)
Supplement: Supplementary file 1 — Additional file 1: Table S1. Criteriafor the liver disease at baseline. Table S2. Characteristics of 6 NAFLD-associated SNPs in the UK Biobank. Table S3. UK Biobank participants characteristics by lifetime cumulativenight shift work duration. Table S4. Average consecutive night shifts and risk of NAFLD. Table S5. Subgroup analyses in current night shift work and NAFLD. Table S6. Subgroup analyses in lifetime duration of night shift work and NAFLD. Table S7. Subgroup analyses in average lifetime night shift frequency and NAFLD. Table S8. Subgroup analyses in average length of each night shift and NAFLD. Table S9. Subgroup analyses in average consecutive night shifts and NAFLD. Table S10. Sensitivity analysis. Figure S1. Associations of night shift work with incident NAFLD by genetic risk. [file 12889_2023_16204_MOESM1_ESM.docx]

**Association between night shift work and NAFLD: a prospective analysis of 281,280 UK Biobank participants**

**(Night shift work and NAFLD)**

Hangkai Huang^1,†^, Zhening Liu^1,†^, Jiarong Xie^1,2,3,†^, Chengfu Xu^1,3,*^

^1^ Department of Gastroenterology, the First Affiliated Hospital, Zhejiang University School of Medicine, Hangzhou, China.

^2^ Department of Gastroenterology, Ningbo First Hospital, Ningbo, China.

^3^Zhejiang Provincial Clinical Research Center for Digestive Diseases, Hangzhou, China.

***Corresponding authors:**

Dr. Chengfu Xu, Department of Gastroenterology, the First Affiliated Hospital, Zhejiang University School of Medicine. No. 79 Qingchun Road, Hangzhou 310003, China. Phone: 0086-571-87236863; E-mail: [xiaofu@zju.edu.cn](mailto:xiaofu@zju.edu.cn)

^†^ Hangkai Huang, Zhening Liu and Jiarong Xie contributed equally to this work.

**Supplementary Table S1. Criteria for the liver disease at baseline**

| **ICD-10 (data field 41270)** | | **Self-reported (data field 20002)** | |
| --- | --- | --- | --- |
| **Code** | **Description** | **Code** | **Description** |
| K76.0 | Fatty (change of) liver, not elsewhere classified | 1136 | Liver/biliary/pancreas problem |
| K75.8 | Other specified inflammatory liver diseases | 1141 | Oesophageal varices |
| K74.0 | Hepatic fibrosis | 1155 | Hepatitis |
| K74.1 | Hepatic sclerosis | 1156 | Infective/viral hepatitis |
| K74.2 | Hepatic fibrosis with hepatic sclerosis | 1157 | Non-infective hepatitis |
| K74.6 | Other and unspecified cirrhosis of liver | 1158 | Liver failure/cirrhosis |
| K76.6 | Portal hypertension | 1159 | Bile duct disease |
| K76.7 | Hepatorenal syndrome | 1408 | Alcohol dependency |
| I85.0 | Oesophageal varices with bleeding | 1506 | Primary biliary cirrhosis |
| I85.9 | Oesophageal varices without bleeding | 1507 | Haemochromatosis |
| I86.4 | Gastric varices | 1578 | Hepatitis a |
| I98.2 | Oesophageal varices in diseases classified elsewhere | 1579 | Hepatitis b |
| I98.3 | Oesophageal varices with bleeding in diseases classified elsewhere | 1580 | Hepatitis c |
| R18 | Ascites | 1581 | Hepatitis d |
| Z94.4 | Liver transplant | 1582 | Hepatitis e |
| C22.0 | Liver cell carcinoma | 1604 | Alcoholic liver disease / alcoholic cirrhosis |

**Supplementary Table S2. Characteristics of 6 NAFLD-associated SNPs in the UK Biobank**

| **RSID** | **Chromosome** | **Position** | **Effect allele** | **Non-effect allele** | **Beta** |
| --- | --- | --- | --- | --- | --- |
| rs12077210 | 1 | 65894160 | T | C | 0.394741 |
| rs2068834 | 2 | 27839539 | C | T | 0.263902 |
| rs13118664 | 4 | 88239609 | T | A | -0.30111 |
| rs139648192 | 8 | 39914918 | T | C | 0.430483 |
| rs17216588 | 19 | 19664077 | T | C | 0.477476 |
| rs738409 | 22 | 44324727 | G | C | 0.602675 |

**Supplementary Table S3. UK Biobank participants characteristics by lifetime cumulative night shift work duration**

|  | lifetime cumulative night shift work duration | | |  |
| --- | --- | --- | --- | --- |
|  | None  (*n*=57003) | <10 years  (*n*=9843) | ≥10 years  (*n*=8213) | *P* value |
| Male (%) | 41.68 | 52.34 | 58.19 | <0.001 |
| Age (years) | 53.04±6.83 | 52.47±6.94 | 52.44±6.7 | <0.001 |
| White Ethnicity (%) | 97.16 | 96.15 | 95.79 | <0.001 |
| Townsend deprivation index | -1.73±2.78 | -1.49±2.9 | -1.52±2.86 | <0.001 |
| College or university degree (%) | 54.16 | 46.38 | 32.49 | <0.001 |
| Household income (£) |  |  |  | <0.001 |
| <18,000 | 5.41 | 5.58 | 5.28 |  |
| 18,000 to 30,999 | 15.31 | 17.03 | 19.26 |  |
| 31,000 to 51,999 | 29.03 | 29.24 | 33.02 |  |
| 52,000 to 100,000 | 33.15 | 31.55 | 27.86 |  |
| >100,000 | 10.2 | 10.26 | 8.28 |  |
| Physical activity (%) |  |  |  | <0.001 |
| Inadequate | 18.33 | 16.76 | 13.16 |  |
| Moderate | 48.92 | 46.48 | 40.48 |  |
| Vigorous | 19.45 | 24.57 | 30.67 |  |
| Smoking status (%) |  |  |  | <0.001 |
| Never | 62.33 | 54.72 | 54.22 |  |
| Previous | 30.97 | 36.3 | 35.93 |  |
| Current | 6.71 | 8.98 | 9.85 |  |
| Alcohol consumption (%) |  |  |  | <0.001 |
| Never or special occasions only | 12.92 | 12.97 | 15.13 |  |
| 1 to 3 times/month | 10.99 | 12.5 | 12.72 |  |
| 1 to 4 times/week | 54.04 | 53.93 | 53.21 |  |
| Daily or almost daily | 22.05 | 20.6 | 18.93 |  |
| Body mass index (kg/m^2^) | 26.46±4.48 | 27.14±4.66 | 27.79±4.76 | <0.001 |
| Hypertension (%) | 43.39 | 44.24 | 47.91 | <0.001 |
| Diabetes (%) | 2.69 | 3.87 | 4.2 | <0.001 |

Values are indicated in means (±standard deviation, SD), or percentages (%), and examined by one-way ANOVA or chi-square test.

**Supplementary Table S4. Average consecutive night shifts and risk of NAFLD**

| Variables | Average consecutive night shifts during night shift periods | | | | *P*_trend_ | Mediation effect (%)  (95% CI) |
| --- | --- | --- | --- | --- | --- | --- |
|  | None | 1 shift | 2-5 shifts | >5 shifts |  |  |
| Case/Sample | 333/57003 | 20/2704 | 101/10593 | 48/4759 |  |  |
| Incidence rate (/100,000 person years) | 48.29 | 61.13 | 78.96 | 83.74 |  |  |
| Model 1 | 1.00 | 1.25 (0.79–1.96) | 1.60 (1.28–2.00) | 1.70 (1.25–2.30) | <0.001 |  |
| Model 2 | 1.00 | 1.20 (0.77–1.89) | 1.42 (1.13–1.78) | 1.53 (1.13–2.08) | <0.001 |  |
| Model 3 | 1.00 | 1.06 (0.68–1.66) | 1.37 (1.10–1.70) | 1.50 (1.12–2.01) | <0.001 |  |
| Mediation model | 1.00 | 1.05 (0.67–1.64) | 1.24 (0.99–1.54) | 1.33 (1.00–1.79) | 0.025 | 28.5 (14.0–49.4) *P*<0.001 |

Model 1 was adjusted for age, sex, and ethnicity (white or others).

Model 2 was model 1 plus further adjustment for Townsend deprivation index, education level (university/college degree or others), household income (less than £18,000, £18,000 to £30,999, £31,000 to £51,999, £52,000 to £100,000, greater than £100,000, or do not know/prefer not to answer), self-reported smoking status (never, former or current smoker), self-reported frequency of alcohol intake (daily/almost daily, 1-4 times a week, 1-3 times a month, or special occasions only/never), and physical activity level (<600, 600-3000, >3000 MET minutes per week, or missing).

Model 3 was model 2 plus further adjustment for diabetes, hypertension, and PRS for NAFLD.
Mediation model was adjusted for BMI separately in addition to model 3 and the mediation effect was calculated.

**Supplementary Table S5. Subgroup analyses in current night shift work and NAFLD**

| Subgroups | Current night shift work | | |  |  |
| --- | --- | --- | --- | --- | --- |
|  | Never/rarely night shifts | Some night shifts | Usual/permanent night shifts |  | *P*_interaction_ |
| Age |  |  |  |  | 0.947 |
| ≤60 years | 1 (reference) | 1.12 (0.95–1.33) | 1.22 (1.02–1.45) |  |  |
| >60 years | 1 (reference) | 0.94 (0.61–1.44) | 1.35 (0.93–1.95) |  |  |
| Gender |  |  |  |  | 0.993 |
| Male | 1 (reference) | 1.13 (0.93–1.38) | 1.30 (1.06–1.59) |  |  |
| Female | 1 (reference) | 1.08 (0.84–1.39) | 1.18 (0.91–1.52) |  |  |
| Household income |  |  |  |  | 0.259 |
| <31000 £/y | 1 (reference) | 1.09 (0.86–1.39) | 1.15 (0.90–1.46) |  |  |
| ≥31000 £/y | 1 (reference) | 1.17 (0.93–1.48) | 1.38 (1.08–1.77) |  |  |
| Townsend index |  |  |  |  | 0.686 |
| Below median | 1 (reference) | 1.01 (0.75–1.36) | 1.18 (0.86–1.60) |  |  |
| Above median | 1 (reference) | 1.19 (0.98–1.45) | 1.30 (1.06–1.58) |  |  |
| Education level |  |  |  |  | 0.388 |
| Others | 1 (reference) | 1.14 (0.96–1.35) | 1.27 (1.07–1.50) |  |  |
| College | 1 (reference) | 0.95 (0.65–1.41) | 1.07 (0.67–1.73) |  |  |
| Smoking |  |  |  |  | 0.636 |
| Never | 1 (reference) | 1.11 (0.90–1.38) | 1.30 (1.05–1.61) |  |  |
| Current or previous | 1 (reference) | 1.07 (0.85–1.35) | 1.16 (0.91–1.47) |  |  |
| Activity |  |  |  |  | 0.196 |
| Below median | 1 (reference) | 1.06 (0.86–1.30) | 1.13 (0.91–1.41) |  |  |
| Above median | 1 (reference) | 1.16 (0.91–1.47) | 1.39 (1.10–1.76) |  |  |
| Hypertension |  |  |  |  | 0.747 |
| No | 1 (reference) | 0.92 (0.69–1.23) | 1.21 (0.92–1.60) |  |  |
| Yes | 1 (reference) | 1.19 (0.99–1.43) | 1.25 (1.03–1.52) |  |  |
| Diabetes |  |  |  |  | 0.160 |
| No | 1 (reference) | 1.08 (0.91–1.28) | 1.28 (1.08–1.52) |  |  |
| Yes | 1 (reference) | 1.21 (0.83–1.76) | 1.07 (0.71–1.61) |  |  |

Model was fully adjusted.

**Supplementary Table S6. subgroup analyses in lifetime duration of night shift work and NAFLD**

|  | Lifetime duration of night shift work | | | | |
| --- | --- | --- | --- | --- | --- |
| Subgroups | None | <10 years | ≥10 years |  | *P*_interaction_ |
| Age |  |  |  |  | 0.093 |
| ≤60 years | 1 (reference) | 1.27 (0.98–1.64) | 1.36 (1.04–1.77) |  |  |
| >60 years | 1 (reference) | 1.06 (0.59–1.91) | 2.28 (1.40–3.72) |  |  |
| Gender |  |  |  |  | 0.236 |
| Male | 1 (reference) | 1.35 (0.98–1.86) | 1.73 (1.28–2.34) |  |  |
| Female | 1 (reference) | 1.11 (0.78–1.58) | 1.25 (0.86–1.83) |  |  |
| Household income |  |  |  |  | 0.643 |
| <31000 £/y | 1 (reference) | 1.00 (0.61–1.66) | 1.40 (0.88–2.21) |  |  |
| ≥31000 £/y | 1 (reference) | 1.27 (0.96–1.69) | 1.49 (1.12–1.99) |  |  |
| Townsend index |  |  |  |  | 0.189 |
| Below median | 1 (reference) | 1.76 (1.26–2.44) | 1.65 (1.15–2.36) |  |  |
| Above median | 1 (reference) | 0.92 (0.64–1.33) | 1.37 (0.98–1.91) |  |  |
| Education level |  |  |  |  | 0.838 |
| Others | 1 (reference) | 1.08 (0.79–1.48) | 1.54 (1.17–2.03) |  |  |
| College | 1 (reference) | 1.49 (1.04–2.13) | 1.32 (0.83–2.10) |  |  |
| Smoking |  |  |  |  | 0.080 |
| Never | 1 (reference) | 1.30 (0.94–1.79) | 1.90 (1.40–2.58) |  |  |
| Current or previous | 1 (reference) | 1.17 (0.83–1.65) | 1.11 (0.77–1.61) |  |  |
| Activity |  |  |  |  | 0.158 |
| Below median | 1 (reference) | 1.40 (1.06–1.86) | 1.66 (1.24–2.21) |  |  |
| Above median | 1 (reference) | 0.95 (0.62–1.45) | 1.27 (0.87–1.87) |  |  |
| Hypertension |  |  |  |  | 0.610 |
| No | 1 (reference) | 1.14 (0.78–1.66) | 1.67 (1.16–2.41) |  |  |
| Yes | 1 (reference) | 1.29 (0.96–1.75) | 1.44 (1.07–1.94) |  |  |
| Diabetes |  |  |  |  | 0.584 |
| No | 1 (reference) | 1.18 (0.92–1.53) | 1.52 (1.18–1.94) |  |  |
| Yes | 1 (reference) | 1.46 (0.78–2.72) | 1.66 (0.85–3.23) |  |  |

Model was fully adjusted.

**Supplementary Table S7. Subgroup analyses in average lifetime night shift frequency and NAFLD**

| Subgroups | Average lifetime night shift frequency | | | | |
| --- | --- | --- | --- | --- | --- |
|  | None | <8/month | ≥8/month |  | *P*_interaction_ |
| Age |  |  |  |  | 0.226 |
| ≤60 years | 1 (reference) | 1.26 (0.98–1.64) | 1.36 (1.04–1.77) |  |  |
| >60 years | 1 (reference) | 1.31 (0.74–2.31) | 1.86 (1.14–3.03) |  |  |
| Gender |  |  |  |  | 0.532 |
| Male | 1 (reference) | 1.49 (1.09–2.04) | 1.57 (1.16–2.14) |  |  |
| Female | 1 (reference) | 1.04 (0.72–1.50) | 1.33 (0.92–1.92) |  |  |
| Household income |  |  |  |  | 0.482 |
| <31000 £/y | 1 (reference) | 1.08 (0.66–1.79) | 1.29 (0.82–2.04) |  |  |
| ≥31000 £/y | 1 (reference) | 1.23 (0.92–1.64) | 1.54 (1.16–2.05) |  |  |
| Townsend index |  |  |  |  | 0.062 |
| Below median | 1 (reference) | 1.46 (1.03–2.08) | 2.00 (1.43–2.80) |  |  |
| Above median | 1 (reference) | 1.16 (0.82–1.63) | 1.11 (0.78–1.58) |  |  |
| Education level |  |  |  |  | 0.636 |
| Others | 1 (reference) | 1.13 (0.83–1.54) | 1.49 (1.13–1.97) |  |  |
| College | 1 (reference) | 1.52 (1.06–2.19) | 1.29 (0.83–2.03) |  |  |
| Smoking |  |  |  |  | 0.924 |
| Never | 1 (reference) | 1.66 (1.22–2.25) | 1.48 (1.07–2.04) |  |  |
| Current or previous | 1 (reference) | 0.89 (0.61–1.30) | 1.45 (1.03–2.03) |  |  |
| Activity |  |  |  |  | 0.309 |
| Below median | 1 (reference) | 1.47 (1.11–1.95) | 1.57 (1.17–2.09) |  |  |
| Above median | 1 (reference) | 0.94 (0.62–1.45) | 1.27 (0.87–1.87) |  |  |
| Hypertension |  |  |  |  | 0.832 |
| No | 1 (reference) | 1.30 (0.89–1.88) | 1.45 (1.00–2.11) |  |  |
| Yes | 1 (reference) | 1.28 (0.94–1.73) | 1.46 (1.08–1.96) |  |  |
| Diabetes |  |  |  |  | 0.448 |
| No | 1 (reference) | 1.22 (0.95–1.58) | 1.46 (1.14–1.88) |  |  |
| Yes | 1 (reference) | 1.71 (0.91–3.19) | 1.39 (0.72–2.69) |  |  |

Model was fully adjusted.

**Supplementary Table S8. Subgroup analyses in average length of each night shift and NAFLD**

| Subgroups | Average length of each night shift during night shift periods | | | | |  |
| --- | --- | --- | --- | --- | --- | --- |
|  | None | <8 hours | 8-12 hours | >12 hours |  | *P*_interaction_ |
| Age |  |  |  |  |  | 0.891 |
| ≤60 years | 1 (reference) | 0.93 (0.65–1.34) | 1.40 (1.05–1.87) | 1.57 (1.18–2.09) |  |  |
| >60 years | 1 (reference) | 2.03 (1.21–3.39) | 1.32 (0.67–2.59) | 1.25 (0.60–2.62) |  |  |
| Gender |  |  |  |  |  | 0.012 |
| Male | 1 (reference) | 1.06 (0.72–1.56) | 1.54 (1.07–2.22) | 2.20 (1.57–3.07) |  |  |
| Female | 1 (reference) | 1.48 (0.94–2.33) | 1.24 (0.83–1.84) | 0.88 (0.54–1.42) |  |  |
| Household income |  |  |  |  |  | 0.211 |
| <31000 £/y | 1 (reference) | 1.35 (0.79–2.30) | 1.03 (0.58–1.83) | 1.21 (0.67–2.17) |  |  |
| ≥31000 £/y | 1 (reference) | 1.05 (0.71–1.54) | 1.37 (0.98–1.91) | 1.70 (1.24–2.33) |  |  |
| Townsend index |  |  |  |  |  | 0.099 |
| Below median | 1 (reference) | 1.46 (0.95–2.26) | 1.68 (1.13–2.49) | 1.98 (1.35–2.91) |  |  |
| Above median | 1 (reference) | 0.93 (0.60–1.44) | 1.23 (0.83–1.81) | 1.24 (0.83–1.87) |  |  |
| Education level |  |  |  |  |  | 0.873 |
| Others | 1 (reference) | 1.21 (0.85–1.72) | 1.18 (0.84–1.65) | 1.60 (1.15–2.23) |  |  |
| College | 1 (reference) | 1.04 (0.60–1.81) | 1.87 (1.22–2.89) | 1.39 (0.87–2.21) |  |  |
| Smoking |  |  |  |  |  | 0.141 |
| Never | 1 (reference) | 1.39 (0.95–2.03) | 1.41 (0.97–2.04) | 1.96 (1.38–2.78) |  |  |
| Current or previous | 1 (reference) | 0.92 (0.57–1.49) | 1.37 (0.93–2.01) | 1.11 (0.73–1.70) |  |  |
| Activity |  |  |  |  |  | 0.203 |
| Below median | 1 (reference) | 1.42 (1.00–2.01) | 1.45 (1.03–2.03) | 1.69 (1.22–2.34) |  |  |
| Above median | 1 (reference) | 0.80 (0.46–1.38) | 1.26 (0.82–1.94) | 1.26 (0.79–2.01) |  |  |
| Hypertension |  |  |  |  |  | 0.935 |
| No | 1 (reference) | 1.27 (0.80–2.02) | 1.33 (0.86–2.04) | 1.51 (0.99–2.31) |  |  |
| Yes | 1 (reference) | 1.13 (0.77–1.65) | 1.42 (1.01–1.99) | 1.56 (1.10–2.20) |  |  |
| Diabetes |  |  |  |  |  | 0.458 |
| No | 1 (reference) | 1.06 (0.77–1.48) | 1.39 (1.05–1.84) | 1.56 (1.17–2.08) |  |  |
| Yes | 1 (reference) | 1.84 (0.92–3.68) | 1.35 (0.59–3.08) | 1.42 (0.66–3.06) |  |  |

Model was fully adjusted.

**Supplementary Table S9. Subgroup analyses in average consecutive night shifts and NAFLD**

| Subgroups | Average Consecutive night shifts during night shift periods | | | | |  |
| --- | --- | --- | --- | --- | --- | --- |
|  | None | 1 shift | 2-5 shifts | >5 shifts |  | *P*_interaction_ |
| Age |  |  |  |  |  | 0.287 |
| ≤60 years | 1 (reference) | 1.12 (0.69–1.82) | 1.29 (1.01–1.65) | 1.46 (1.05–2.03) |  |  |
| >60 years | 1 (reference) | 0.82 (0.26–2.60) | 1.77 (1.09–2.89) | 1.67 (0.89–3.12) |  |  |
| Gender |  |  |  |  |  | 0.257 |
| Male | 1 (reference) | 1.18 (0.64–2.18) | 1.53 (1.15–2.05) | 1.71 (1.18–2.48) |  |  |
| Female | 1 (reference) | 0.95 (0.49–1.85) | 1.19 (0.85–1.68) | 1.26 (0.78–2.05) |  |  |
| Household income |  |  |  |  |  | 0.615 |
| <31000 £/y | 1 (reference) | 1.08 (0.44–2.68) | 1.07 (0.68–1.69) | 1.56 (0.88–2.76) |  |  |
| ≥31000 £/y | 1 (reference) | 1.04 (0.59–1.81) | 1.43 (1.09–1.87) | 1.44 (1.00–2.06) |  |  |
| Townsend index |  |  |  |  |  | 0.079 |
| Below median | 1 (reference) | 1.45 (0.77–2.76) | 1.76 (1.27–2.42) | 1.73 (1.12–2.68) |  |  |
| Above median | 1 (reference) | 0.99 (0.52–1.88) | 1.09 (0.79–1.52) | 1.30 (0.84–2.00) |  |  |
| Education level |  |  |  |  |  | 0.971 |
| Others | 1 (reference) | 0.85 (0.44–1.66) | 1.31 (1.00–1.72) | 1.52 (1.07–2.17) |  |  |
| College | 1 (reference) | 1.35 (0.73–2.48) | 1.44 (0.98–2.11) | 1.46 (0.86–2.48) |  |  |
| Smoking |  |  |  |  |  | 0.225 |
| Never | 1 (reference) | 1.26 (0.70–2.26) | 1.58 (1.18–2.13) | 1.70 (1.16–2.50) |  |  |
| Current or previous | 1 (reference) | 0.86 (0.43–1.75) | 1.14 (0.82–1.59) | 1.31 (0.83–2.05) |  |  |
| Activity |  |  |  |  |  | 0.147 |
| Below median | 1 (reference) | 1.17 (0.68–2.00) | 1.57 (1.20–2.05) | 1.60 (1.12–2.30) |  |  |
| Above median | 1 (reference) | 0.89 (0.39–2.03) | 1.06 (0.73–1.55) | 1.32 (0.81–2.15) |  |  |
| Hypertension |  |  |  |  |  | 0.595 |
| No | 1 (reference) | 0.93 (0.43–1.98) | 1.31 (0.92–1.87) | 1.77 (1.14–2.76) |  |  |
| Yes | 1 (reference) | 1.16 (0.66–2.04) | 1.41 (1.07–1.86) | 1.37 (0.93–2.02) |  |  |
| Diabetes |  |  |  |  |  | 0.692 |
| No | 1 (reference) | 0.87 (0.51–1.48) | 1.41 (1.12–1.77) | 1.43 (1.04–1.97) |  |  |
| Yes | 1 (reference) | 2.51 (1.05–6.02) | 1.09 (0.55–2.15) | 2.05 (0.98–4.26) |  |  |

Model was fully adjusted.

**Supplemental Table S10.** **Sensitivity analysis**

| Subgroups | Exclude the first 2 years of follow-up | Exclude excessive alcohol intake | Exclude poor sleep pattern | Widen the definition of endpoint |
| --- | --- | --- | --- | --- |
| Current work |  |  |  |  |
| Never/rarely night shifts | 1 (ref) | 1 (ref) | 1 (ref) | 1 (ref) |
| Some night shifts | 1.12 (0.95–1.31) | 1.17 (0.95–1.43) | 1.20 (1.02–1.41) | 1.02 (0.91–1.13) |
| Usual/permanent night shifts | 1.32 (1.13–1.56) | 1.22 (0.99–1.50) | 1.26 (1.05–1.51) | 1.22 (1.09–1.36) |
| *P* for trend | 0.004 | 0.003 | 0.011 | 0.020 |
| Lifetime |  |  |  |  |
| Duration |  |  |  |  |
| None | 1 (ref) | 1 (ref) | 1 (ref) | 1 (ref) |
| <10 years | 1.28 (1.00–1.62) | 1.51 (1.12–2.04) | 1.27 (0.99–1.64) | 1.17 (1.00–1.36) |
| ≥10 years | 1.54 (1.22–1.96) | 1.58 (1.15–2.16) | 1.71 (1.34–2.18) | 1.25 (1.06–1.46) |
| *P* for trend | 0.002 | 0.005 | <0.001 | 0.003 |
| Frequency |  |  |  |  |
| None | 1 (ref) | 1 (ref) | 1 (ref) | 1 (ref) |
| <8/month | 1.30 (1.02–1.65) | 1.41 (1.03–1.92) | 1.26 (0.98–1.63) | 1.18 (1.02–1.38) |
| ≥8/month | 1.51 (1.19–1.91) | 1.69 (1.25–2.28) | 1.71 (1.34–2.16) | 1.22 (1.04–1.43) |
| *P* for trend | <0.001 | 0.002 | <0.001 | 0.006 |
| Length |  |  |  |  |
| None | 1 (ref) | 1 (ref) | 1 (ref) | 1 (ref) |
| <8 hours | 1.06 (0.67–1.68) | 1.31 (0.89–1.94) | 1.24 (0.91–1.69) | 1.15 (0.91–1.47) |
| 8-12 hours | 1.42 (1.14–1.78) | 1.67 (1.19–2.34) | 1.55 (1.18–2.05) | 1.24 (0.99–1.57) |
| >12 hours | 1.53 (1.14–2.07) | 1.62 (1.13–2.32) | 1.62 (1.22–2.15) | 1.39 (1.05–1.84) |
| *P* for trend | <0.001 | 0.004 | <0.001 | 0.001 |
| Consecutiveness |  |  |  |  |
| None | 1 (ref) | 1 (ref) | 1 (ref) | 1 (ref) |
| 1 shift | 1.06 (0.67–1.68) | 1.42 (0.82–2.44) | 1.15 (0.72–1.83) | 1.17 (0.82–1.67) |
| 2-5 shifts | 1.42 (1.14–1.78) | 1.51 (1.13–2.01) | 1.50 (1.19–1.88) | 1.23 (1.02–1.49) |
| >5 shifts | 1.53 (1.14–2.07) | 1.69 (1.15–2.48) | 1.60 (1.18–2.18) | 1.30 (1.06–1.59) |
| *P* for trend | 0.001 | 0.003 | <0.001 | 0.002 |

Model was fully adjusted.

**
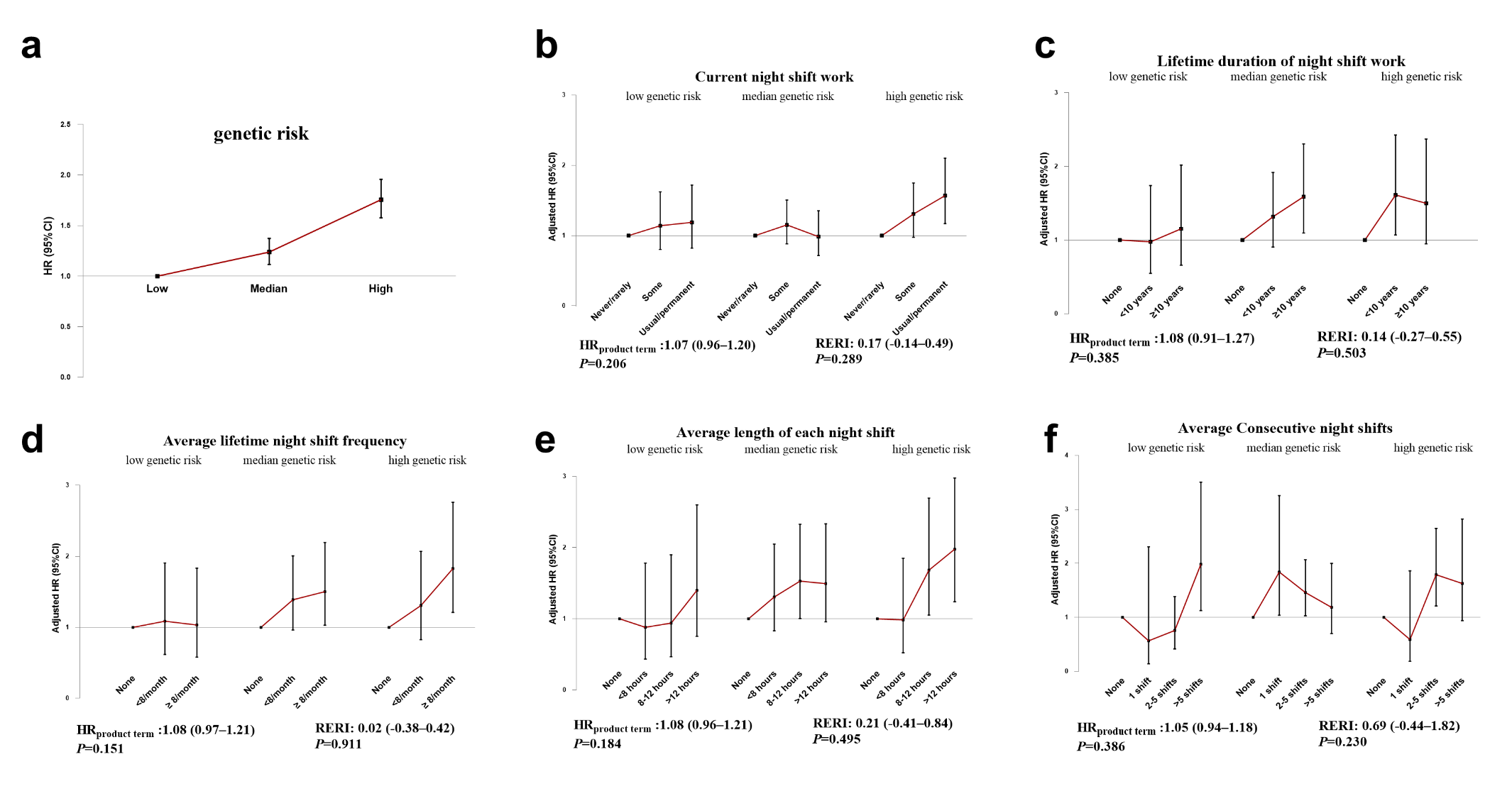
Supplementary Figure S1. Associations of night shift work with incident NAFLD by genetic risk**

(a) Association of PRS groups [high (highest quartile), median (mid-two quartiles), or low (lowest quartile)] with the risk of NAFLD.

(b-f) Association of current night shift work (b), lifetime duration of night shift work (c), average lifetime night shift frequency (d), average length of each night shift (e), and average consecutive night shifts (f) with the risk of NAFLD according to PRS groups.

Hazard ratios were adjusted for age, sex, Townsend deprivation index, education level, household income, self-reported smoking status, self-reported frequency of alcohol intake, physical activity level, diabetes and hypertension.

Multiplicative interaction was evaluated using overall hazard ratios for the product term. Additive interaction was evaluated using relative excess risk due to interaction (RERI) between the genetic risk (low vs. high) and night shift work (none vs. the highest group).
